# IFNβ autocrine feedback is required to sustain TLR induced production of MCP-1 in macrophages

**DOI:** 10.1016/j.febslet.2013.03.025

**Published:** 2013-05-21

**Authors:** Michael J. Pattison, Kirsty F. MacKenzie, Suzanne E. Elcombe, J. Simon C. Arthur

**Affiliations:** a MRC Protein Phosphorylation Unit, Wellcome Trust Building, College of Life Sciences, University of Dundee, Dundee DD1 5EH, Scotland; b Division of Cell Signaling and Immunology, Wellcome Trust Building, College of Life Sciences, University of Dundee, Dundee DD1 5EH, Scotland

**Keywords:** MCP-1, IL-10, MAPK, TLR, Signaling

## Abstract

Chemokines, including MCP-1, are crucial to mounting an effective immune response due to their ability to recruit other immune cells. We show that sustained LPS or poly(I:C)-stimulated MCP-1 production requires an IFNβ-mediated feedback loop. Consistent with this, exogenous IFNβ was able to induce MCP-1 transcription in the absence of other stimuli. Blocking IFNβ signaling with Ruxolitinib, a JAK inhibitor, inhibited MCP-1 transcription. The MCP-1 promoter contains potential STAT binding sites and we demonstrate that STAT1 is recruited upon IFNβ stimulation. Furthermore we find that IL-10 knockout increases MCP-1 production in response to LPS, which may reflect an ability of IL-10 to repress IFNβ production. Overall, these results show the importance of the balance between IFNβ and IL-10 in the regulation of MCP-1.

## Highlights

• MCP-1 mRNA levels and protein secretion in macrophages are induced by TLR activation.
• In response to LPS, the initial induction of MCP-1 mRNA is IFNβ independent.
• The sustained production of MCP-1 by LPS requires an IFNβ mediated feedback loop.
• The sustained production of MCP-1 by poly IC also requires IFNβ.

## 1 Introduction

The immune system has developed to recognise pathogens and trigger an inflammatory response. Macrophages and other innate immune cells recognise pathogens via pattern recognition receptors (PRRs) and their activation helps promote the production of chemokines and cytokines [1–3]. Chemokines produced by cells present at the site of infection establish a chemoattractant gradient to recruit other immune cells [4]. Chemokines are small proteins often with 4 conserved cysteine residues that form two disulphide bonds and the position of these cysteine residues has been used to define sub-families of chemokines [5]. Monocyte chemotactic proteins (MCPs) form a subfamily of β-chemokines consisting of 4 proteins (MCP-1, 2, 3 and 4) that share over 60% homology with each other [6].

MCP-1 is encoded by the *ccl2* gene and can be produced by several cell types including macrophages and fibroblasts [7]. MCP-1 acts as chemo-attractant for monocytes as well as some other immune cells such as memory T lymphocytes and natural killer cells [8]. As a result, MCP-1 knockout mice have impaired monocyte recruitment following intraperitoneal thioglycollate administration, induction of delayed-type hypersensitivity models or in response to *Schistosoma mansoni* eggs [9]. MCP-1 can however also contribute to several diseases. For example, MCP-1 promotes allergic inflammation by inducing immune cell infiltration and stimulating histamine release from mast cells and basophils [10]. In inflammatory bowel disease, higher levels of MCP-1 in the mucosa of patients may inhibit the differentiation of monocytes into tolerogenic intestinal macrophages [11]. MCP-1 protein levels are also increased in the plasma of patients with rheumatoid arthritis [12] and diabetes [13]. Changes in MCP-1 expression as a result of nucleotide polymorphisms have been correlated to several diseases. For example, an A-2518G promoter polymorphism is linked to infection; carriers of the AG or GG genotypes were more likely to develop tuberculosis, which was associated with increased levels of MCP-1 but less IL-12p40 [14]. This same polymorphism has also been linked to psoriasis [15]. G-928C and G-362C (which lies in a potential STAT binding site) promoter polymorphisms are associated with an increased risk for carotid atherosclerosis and correlate to increased expression of MCP-1 [16].

Given its multiple roles in disease, how MCP-1 production is controlled is an important question. Several agonists have been found to induce MCP-1 secretion and a range of signalling pathways and transcription factors have been implicated in this process. For example, the induction of MCP-1 expression by LPS in macrophages is dependent on Tpl2/ERK signalling, as has previously been shown using both Tpl2 deficient macrophages and small molecule inhibitors targeting ERK1/2 [17]. TNFα responsive NFκB elements have been identified in the murine MCP-1 promoter and they are also likely to control MCP-1 gene transcription in response to TLR agonists [18]. A role for Sp-1 in promoting the assembly of promoter complexes to drive TNFα induced MCP-1 gene expression has been demonstrated using Sp1−/− embryonic fibroblasts [19]. In B10R cells, a murine macrophage cell line, roles for NFκB, AP-1 and CREB in MCP-1 transcription in response to hydrogen peroxide have been proposed based on small molecule inhibitors and EMSA [20]. Several studies have suggested roles for STATs in the transcription of MCP-1. STATs are activated by JAKs and are responsible for mediating many effects of cytokine signalling. For example, both STAT1 and STAT3 were required for maximal MCP-1 expression in an osteoblast cell line in response to oncostatin M [21]. Furthermore, STAT4 was required for MCP-1 expression in murine mast cells in response to IFNβ [22].

In macrophages, MCP-1 expression can be induced by activation of various PRRs, including members of the Toll-like receptor (TLR) family. The regulation of cytokine production by TLR4 is complicated by the ability of certain LPS induced cytokines to act in a paracrine or autocrine manner to modulate cytokine production. For instance, LPS results in the secretion of IL-10 and IFNβ [23]. IL-10 acts as a negative feedback mechanism and can repress pro-inflammatory cytokine and prostaglandin production while IFNβ helps sustain the LPS induced transcription of IL-10 and IL-6 [24–28]. Interestingly, both IL-10 and IFNβ activate JAK/STAT signalling in macrophages, however despite this, following LPS stimulation IL-10 promotes STAT3 phosphorylation while IFNβ results in STAT1 phosphorylation [29,30]. We show here that while IFNβ is not required for the initial transcription or secretion of MCP-1 in bone marrow derived macrophages (BMDMs), it is required to sustain MCP-1 production in response to both TLR4 and TLR3 activation.

## 2 Materials and methods

### 2.1 Mice

C57/Bl6 wild-type mice were obtained from Charles River Laboratories or bred in house. Mice were maintained under specific pathogen free conditions. Work was carried out in accordance with EU and UK regulations and covered by a UK Home Office project licence. IL-10 and IFNαβR knockout mice have been described previously [31] and were backcrossed on C57/Bl6 mice for at least 12 generations.

### 2.2 Cell culture

Primary bone marrow derived macrophages (BMDMs) were isolated as described previously [32]. Where indicated cells were pre-treated with 0.5 μM Ruxolitinib (JAK inhibitor), 2 μM PD184352 (MEK1/2 inhibitor) or 10 μg/ml IL-10 neutralising antibody. We have previously shown that in macrophages these concentrations block JAK, ERK1/2 or IL-10 signalling respectively [32,33].

### 2.3 Q-pcr

Cells were lysed and total RNA purified using the Qiagen microRNeasy system. Total RNA (0.5–1 μg) was reversed transcribed using iScript (Bio-Rad) and qPCR carried out using sybergreen based detection. Levels of 18s were used as a normalization control, and fold induction calculated as described previously [32]. Primer sequences were: TTTGAATGTGAAGTTGACCCGTAAATC and TCACTGTCACACTGGTCACTCC (MCP-1); GTAACCCGTTGAACCCCATT and CCATCCAATCGGTAGTAGCG (18s); GGAAAAGCAAGAGGAAAGATTGAC and CCACCATCCAGGCGTAGC (IFNβ).

### 2.4 Cytokine measurements

MCP-1 was measured using a Luminex-based assay (Bio-Rad), according to the manufacturers’ protocol. Briefly, cytokines are captured using antibody-coupled beads. A biotinylated detection antibody then binds to the complex followed by a streptavidin-PE reporter complex. Samples are measured on a dual-laser, flow-based microplate reader system. IFNβ was measured by ELISA (Pbl Interferon Source).

### 2.5 Immunoblotting

Immunoblotting was carried out using standard techniques [32]. Phospho STAT2 and 6 antibodies were from Abcam and phospho STAT5 and ERK2 from Cell Signaling Technology.

### 2.6 Chromatin Immunoprecipitation

Cells were stimulated with 500 U/ml IFNβ for 30 min and chromatin immunoprecipitations performed as described previously [34]. Anti-STAT1 and anti-IgG antibodies (Cell Signaling Technology) were used. STAT1 or IgG ChIP DNA from either nn-stimulated or IFN-stimulated cells was analyzed by qPCR to test for the presence of STAT1 target sequences in the promoter regions of *CCL2 (Forward-* CACTTCCTGGAAACACCCGA and Reverse- CTTGGTGCCAAGGAGTAGCA) and a region in the *GAPDH* with no known STAT binding site (Forward- AGTGCCAGCCTCGTCCCGTAGACAAAATG and Reverse- AAGTGGGCCCCGGCCTTCTCCAT). ChIP data was calculated as percentage of input DNA for each sample.

## 3 Results

**Fig. 1:**
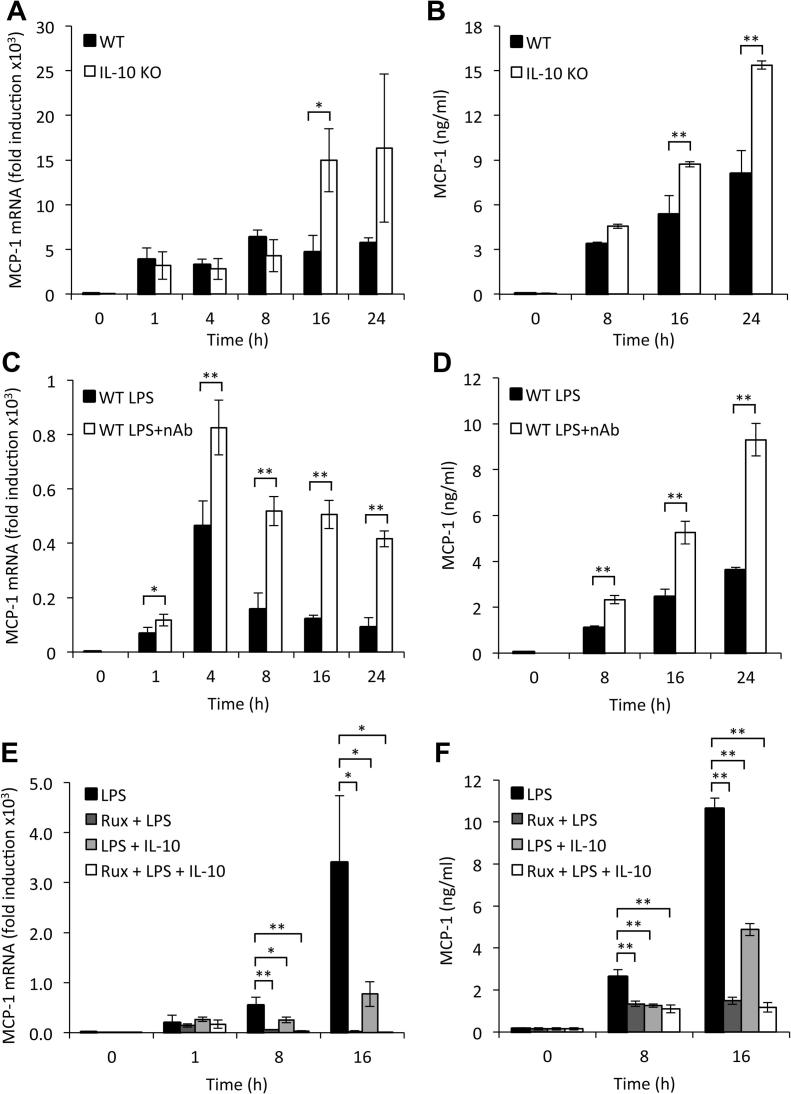
Blocking IL-10 increases MCP-1 in BMDMs. (A and B) BMDMs were isolated from wild-type and IL-10 KO mice and then stimulated with 100 ng/ml LPS for the times indicated and MCP-1 mRNA levels (determined by Q-PCR) (A) or the levels of MCP-1 protein secreted (B) were determined. (C and D) BMDMs from wild-type mice were treated with 10 μg/ml IL-10 neutralising antibody for 1 h. Cells were then stimulated with 100 ng/ml LPS for the times indicated and MCP-1 mRNA levels (C) or MCP-1 secretion (D) determined. (E and F) BMDMs were isolated from IL-10 KO mice and where indicated treated with 0.5 μM Ruxolitinib for 1 h. Cells were then treated with either 100 ng/ml LPS alone or a combination of both LPS and 100 ng/ml IL-10. MCP-1 mRNA induction (E) or secreted levels of MCP-1 (F) were determined at the indicated times. In all panels error bars represent the standard deviation from independent cultures from 4 mice per genotype. A p value (students t-test) relative to the wild type cells of less than 0.05 is indicated by ^∗^ and less than 0.01 by ^∗∗^.

In response to the TLR4 agonist LPS, BMDMs secrete IL-10 and this sets up a feedback mechanism that inhibits the production of multiple pro-inflammatory mediators [27]. As IL-10 acts via a JAK/STAT pathway and as STATs have been implicated in MCP-1 transcription, we examined the role of IL-10 in LPS induced MCP-1 production. Following LPS stimulation, wild-type BMDMs rapidly induced MCP-1 mRNA and this increased level was maintained over 24 h. Knockout of IL-10 did not affect the initial induction of MCP-1 mRNA. In contrast, at later time points, IL-10 knockout BMDMs had a moderately higher induction of MCP-1 compared to wild-type cells (Fig 1A). The increase in MCP-1 mRNA in the absence of IL-10 translated into elevated secretion of MCP-1 by the IL-10 knockout cells relative to wild-type cells at 16 and 24 h after LPS stimulation (Fig 1B). To confirm these results, LPS stimulation of wild-type cells was carried out in the presence of a neutralizing antibody to IL-10. Similar to the IL-10 knockout, the IL-10 neutralizing antibody did not greatly affect the initial induction of MCP-1 mRNA but did increase MCP-1 mRNA induction and MCP-1 protein secretion at later time points (Fig 1C and D). These results suggest that IL-10 represses MCP-1 production. To confirm that IL-10 could directly repress MCP-1 induction, IL-10 knockout BMDMs were isolated and stimulated with LPS in the presence or absence of exogenous IL-10. The addition of exogenous IL-10 repressed LPS stimulated MCP-1 transcription and MCP-1 protein secretion (Fig 1E and F). IL-10 signals via the kinases JAK1 and Tyk2, the effects of IL-10 should therefore be blocked by JAK inhibitors. We therefore tested the ability of Ruxolitinib, a JAK inhibitor, to block the effect of IL-10 on MCP-1 induction. We have previously shown that Ruxolitinib shows similar IC50s for JAK1, JAK2 and Tyk2 in vitro [32]. In this study we also demonstrated that at the concentration used in Fig 1, Ruxolitinib was able to block both IL-10 induced STAT3 phosphorylation and IFNβ stimulated STAT1 phosphorylation in BMDMs. Addition of exogenous IL-10 resulted in decreased MCP-1 production. This decrease however was not blocked by addition of Ruxolitinib. In fact, Ruxolitinib treatment inhibited MCP-1 secretion or sustained MCP-1 mRNA induction in response to either LPS alone or a combination of LPS and exogenous IL-10. This indicates another JAK or Tyk2 dependent pathway distinct from IL-10 is required for maximal MCP-1 expression (Fig 1F).

**Fig. 2:**
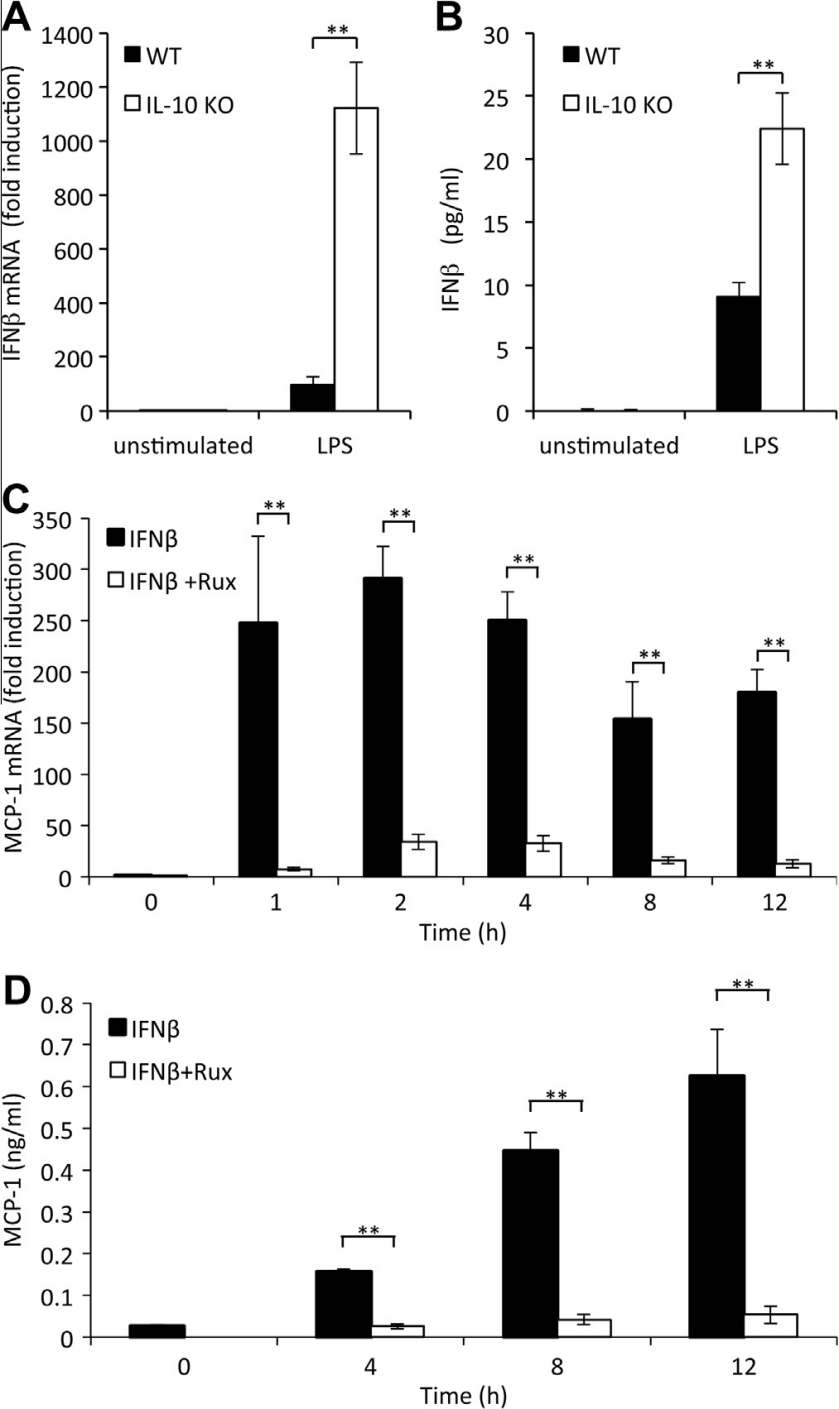
IFNβ can induce MCP-1 transcription in BMDMs. (A and B) BMDMs were isolated from wild-type and IL-10 KO mice and then stimulated with 100 ng/ml LPS for 8 h and IFNβ mRNA levels (A) or IFNβ secretion (B) measured by qPCR or ELISA respectively. (C and D) BMDMs were isolated from wild-type mice and incubated with 0.5 μM Ruxolitinib for 1 h where indicated before stimulation with 500 Units/ml IFNβ for the times indicated. Total RNA was isolated and MCP-1 mRNA levels determined by Q-PCR (C). Alternatively, MCP-1 protein levels secreted into the media were determined (D). Error bars represent the standard deviation from independent cultures from 3 (A and B) or 4 (C and D) mice per genotype. A *P* value (students *t*-test) relative to the no inhibitor conditions of less than 0.05 is indicated by ^∗^ and less than 0.01 by ^∗∗^.

**Fig. 3:**
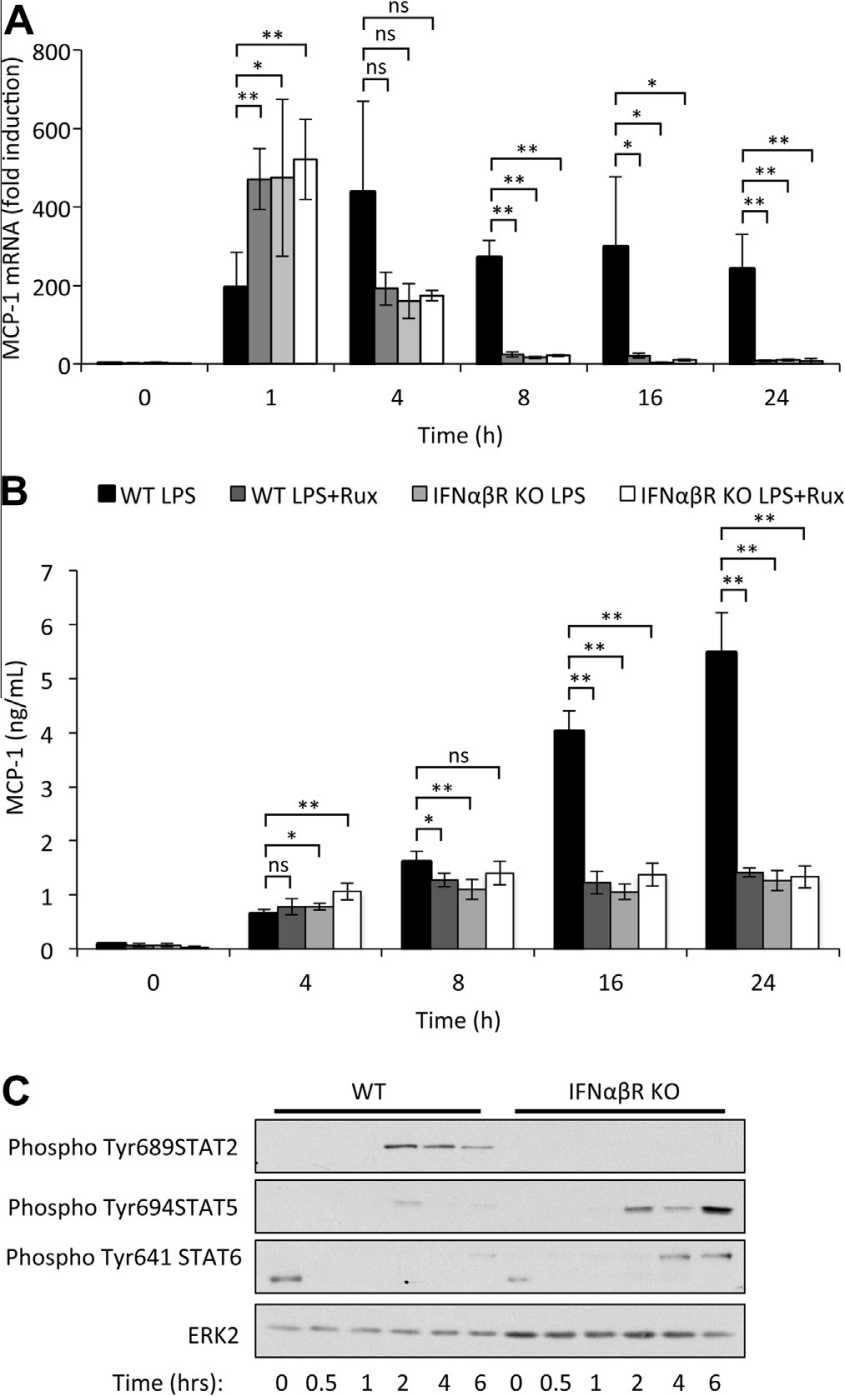
Induction of signaling by IFNβ is required for sustained MCP-1 transcription and secretion. BMDMs were isolated from wild-type or IFNαβR knockout mice and incubated with 0.5 μM Ruxolitinib for 1 h where indicated. Cells were stimulated with 100 ng/ml LPS for the indicated times and MCP-1 mRNA levels (A) or secreted levels were determined (B). The levels of phosphorylated STAT2, 5 and 6 were determined by immunoblotting (C). Error bars represent the standard deviation from independent cultures from 4 mice per genotype. A p value (students t-test) relative to the wild type cells in the absence of Ruxolitinib of less than 0.05 is indicated by ^∗^ and less than 0.01 by ^∗∗^.

IFNβ is secreted by BMDMs following LPS stimulation and is also able to activate JAK1/Tyk2 signaling. IL-10 is known to repress IFNβ induction in response to LPS [35,36], thus the results in Fig 1E could be explained by a requirement for IFNβ for sustained MCP-1 mRNA induction. In line with this, knockout of IL-10 resulted in both increased IFNβ mRNA and IFNβ protein section in response to LPS (Fig. 2A and B). The treatment of BMDMs with exogenous IFNβ demonstrated that IFNβ was sufficient to increase both MCP-1 mRNA levels and secreted MCP-1 protein levels (Fig. 2C and D). Consistent with IFNβ stimulating JAK/STAT signaling, this was inhibited by Ruxolitinib (Fig. 2C and D). To test whether IFNβ was required to maintain LPS induced MCP-1 induction, cells were isolated from mice with a knockout in the type 1 interferon receptor (IFNαβR knockout). IFNαβR knockout macrophages showed a transient increase in MCP-1 transcription compared to wild-type cells following a 1 h stimulation with LPS. The reason for this increase is unknown. Despite this early increase, the knockout cells were unable to sustain MCP-1 mRNA induction resulting in lower levels relative to wild-type cells at later time points (Fig. 3A). As IFNβ requires JAK1 and Tyk2 to activate intracellular signaling, we hypothesized that the effects of an IFNβ feedback loop should be blocked by Ruxolitinib. In confirmation of this, treatment of wild-type cells with Ruxolitinib mirrored the effect of IFNαβR knockout on MCP-1 mRNA induction (Fig. 3A). Ruxolitinib did not have an additive effect in combination with the IFNαβR knockout on MCP-1 mRNA induction, in line with it acting downstream of IFNβ. Consistent with the mRNA results, neither knockout of the IFNαβR nor Ruxolitinib treatment greatly affected the initial secretion of MCP-1 in response to LPS (Fig. 3B). Ruxolitinib did however reduce the secreted levels of MCP-1 at 16 and 24 h from wild-type cells. IFNαβR knockout also reduced MCP-1 secretion relative to wild-type controls at 16 and 24 h (Fig. 3B). As for MCP-1 mRNA, Ruxolitinib did not have an additive with IFNαβR knockout on MCP-1 secretion (Fig. 3B). We have previously shown that IFNβ is required for LPS induced STAT1 but not STAT3 phosphorylation [32]. In addition to STAT1, STAT2 Tyr phosphorylation was dependent on IFNβ signalling, although in contrast the Tyr phosphorylation of STAT5 and 6 was increased in IFNαβR knockout BMDMs (Fig. 3C).

**Fig. 4:**
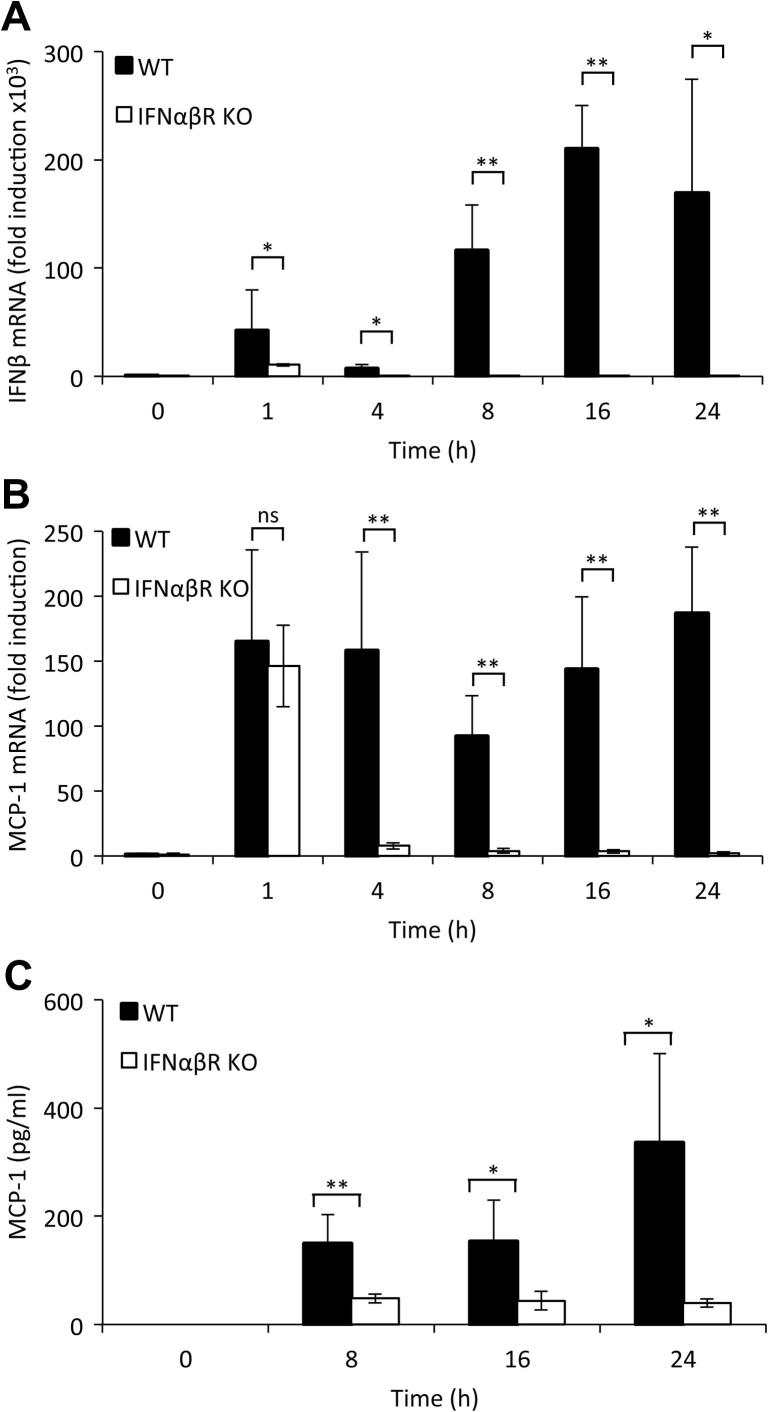
Poly(I:C) induces biphasic IFNβ production which is required for sustained MCP-1 production. BMDMs were isolated from wild-type or IFNαβR knockout mice and stimulated with 10 μg/ml poly(I:C) for the indicated times and IFNβ mRNA (A) or MCP-1 mRNA (B) levels determined by Q-PCR. Alternatively the levels of MCP-1 secreted into the media were determined by Luminex based assay (C). In all panels error bars represent the standard deviation from independent cultures of 4 mice per genotype. A *P* value (students *t*-test) relative to the wild type cells of less than 0.05 is indicated by ^∗^ and less than 0.01 by ^∗∗^.

As TLR3 activation also promotes IFNβ production by macrophages we examined the effect of TLR3 agonist poly(I:C) on MCP-1 production. Consistent with what has been reported previously [37–39], poly(I:C) induces significant IFNβ transcription, which was biphasic in macrophages, with the second wave of IFNβ transcription dependent on type I IFN signalling as it was absent in IFNαβR knockout BMDMs (Fig. 4A). MCP-1 mRNA levels were increased by stimulation with poly(I:C), although to lower levels than seen with LPS stimulation. At 1 h, MCP-1 transcription was independent of type I IFN signaling as it was unaffected by the IFNαβR knockout (Fig. 4B). However, the sustained induction of MCP-1 mRNA was lost in IFNαβR knockout (Fig 4B). Poly(I:C) was also able to induce MCP-1 secretion, and in line with the mRNA results MCP-1 secretion was considerably lower from poly(I:C) stimulated IFNαβR knockout BMDMs relative to wild-type cells (Fig. 4C).

**Fig. 5:**
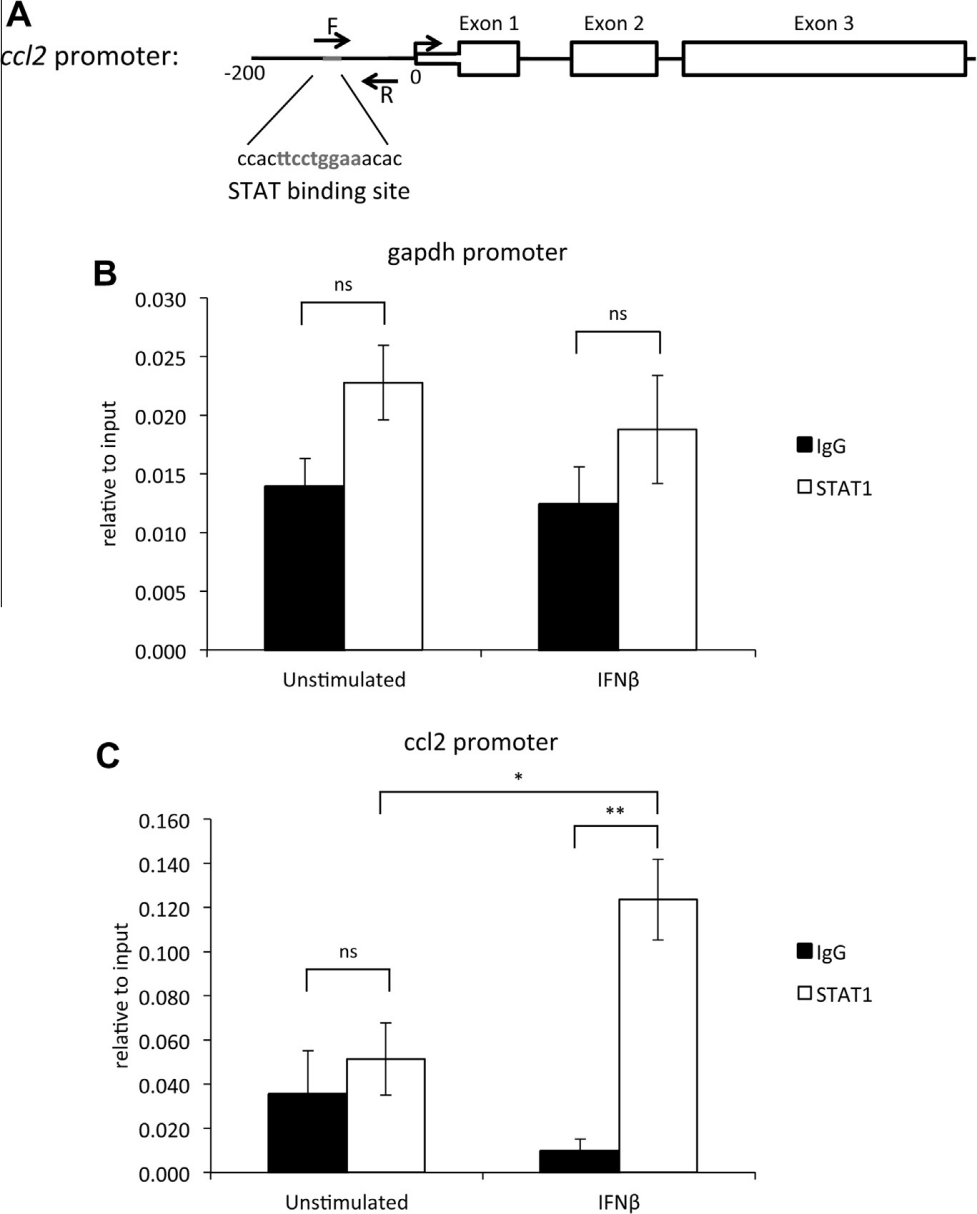
IFNβ stimulation recruits STAT1 to the MCP-1 promoter. To analyse STAT1 recruitment to the MCP-1 promoter by ChIP, qPCR using primers designed to cover a potential STAT-binding site in the *ccl2 (MCP-1)* promoter (A). BMDMs were isolated from wild-type mice and stimulated with 500 U/ml IFNβ for 30 min. Cells were cross-linked and STAT1 or control IgG immunoprecipitations performed. ChIP signals relative to input for a region of GAPDH gene (B) and the STAT binding region of the ccl2 promoter (C) are shown. Error bars represent the s.e.m. of stimulations from 4 independent cultures of BMDMs. A p value (students *t*-test) of less than 0.05 is indicated by ^∗^ and less than 0.01 by ^∗∗^.

Loss of the type I interferon receptor leads to reduced STAT1 [32] and STAT2 phosphorylation (Fig 3C), therefore perhaps loss of activated STAT transcription factors is responsible for the reduced transcription of MCP-1 seen at later time points. Furthermore, a potential STAT binding site is evident within the MCP-1 promoter (Fig. 5A). To investigate a direct role for STAT1 in MCP-1 transcription, primary macrophages were stimulated with IFNβ and STAT1 chromatin immunoprecipitation carried out. Upon stimulation with IFNβ, a marked increase in STAT1 was found at the MCP-1 promoter compared to an IgG negative control and in the unstimulated state with STAT1 and IgG (Fig. 5B and C).

**Fig. 6:**
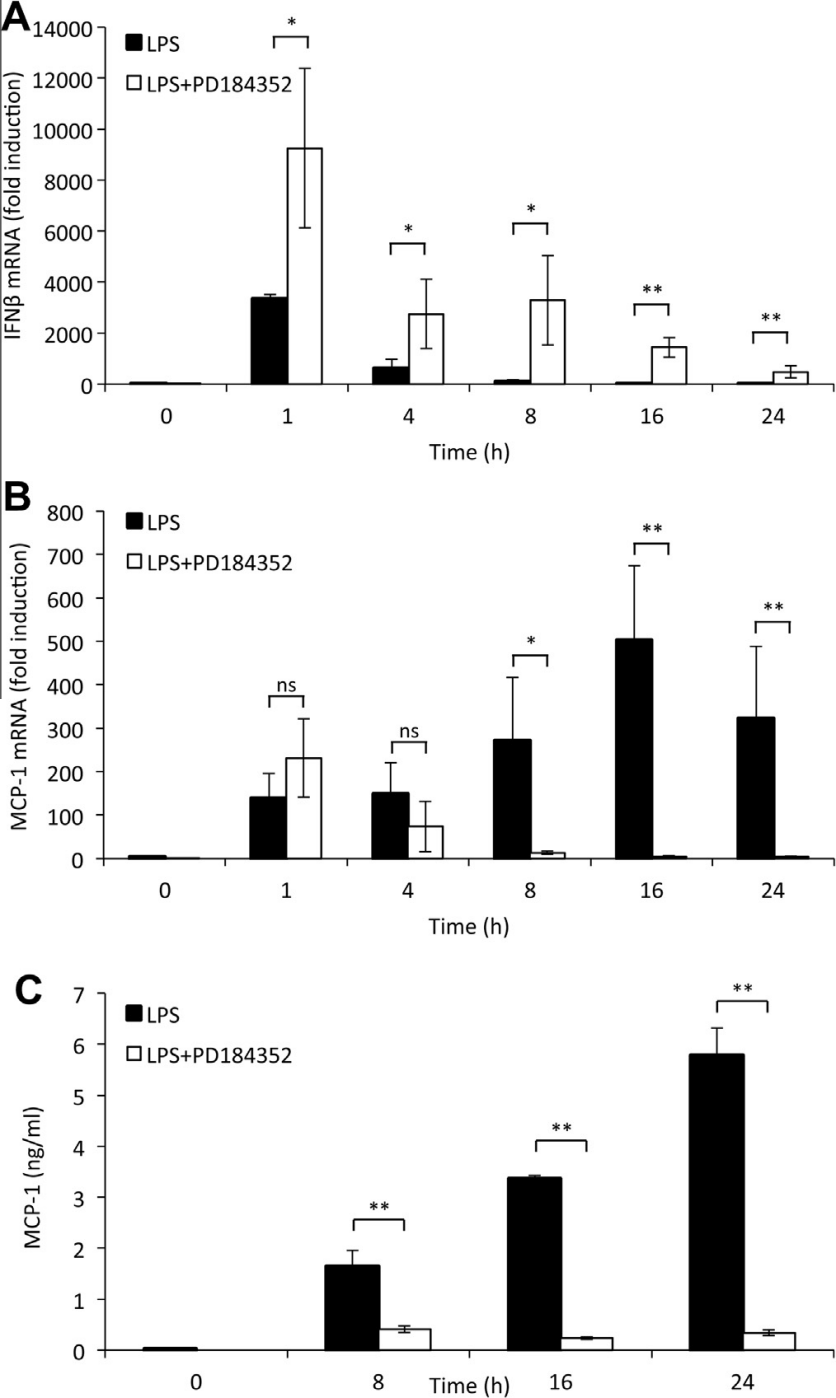
Blocking ERK signaling causes decreased MCP-1 production. BMDMs were isolated from wild-type mice and incubated with 2 μM PD184352 for 1 h where indicated. Cells were stimulated with 100 ng/ml LPS for the indicated times and IFNβ mRNA levels were determined by qPCR (A). Additionally, MCP-1 mRNA (B) and secreted levels of MCP-1 (C) were measured. In all panels error bars represent the standard deviation from independent cultures from 4 mice. A p value (students *t*-test) relative to the cells without PD184352 of less than 0.05 is indicated by ^∗^ and less than 0.01 by ^∗∗^. NS represents a p value greater than 0.5.

The ERK1/2 pathway can inhibit IFNβ transcription resulting in less IFNβ secretion [40]. As a result, blocking ERK1/2 pharmacologically may lead to increased IFNβ production which would then further stimulate MCP-1 production at later time points. Against this, a direct role for ERK1/2 in promoting MCP-1 induction has also been suggested [17]. To investigate this, macrophages were stimulated with LPS for up to 24 h in the presence or absence of 2 μM PD184352, a MEK1/2 inhibitor that blocks the activation of ERK1/2 in response to LPS [33]. Treatment with the ERK1/2 inhibitor lead to significantly increased IFNβ mRNA transcription from 1 h onwards (Fig. 6A). However, this did not lead to increase in MCP-1 transcription (Fig. 6B) or secretion (Fig. 6C). In contrast PD 184352 actually decreased the induction of both secreted MCP-1 protein and MCP-1 mRNA at later time points, an observation consistent with a previous report indicating that ERK1/2 can directly promote MCP-1 production [17].

## 4 Discussion

The sustained production of pro-inflammatory cytokines by TLR agonists is modulated by autocrine signaling. For instance, endogenously produced IL-10 acts to inhibit further pro-inflammatory cytokine production and this is important to keep cytokine production in check and to prevent excessive inflammation [24]. In contrast, IFNβ helps maintain the production of specific cytokines downstream of LPS [41]. The importance of this in vivo is demonstrated by the finding that IFNβ knockout mice are protected from LPS-induced endotoxic shock [42]. Interestingly, despite their different actions on macrophages, both IFNβ and IL-10 signal via JAK1 and Tyk2. Following LPS stimulation however, endogenously produced IL-10 results in STAT3 phosphorylation while IFNβ is responsible for STAT1 phosphorylation [32,33]. The molecular details of this specificity are not clear and specificity is lost if exogenous IL-10 or IFNβ are added at high concentrations [43–45].

We demonstrate here that the sustained production of MCP-1 in macrophages in response to LPS or poly(I:C) requires an IFNβ dependent feedback loop. Additionally, we show that IFNβ can directly stimulate MCP-1 transcription and that Ruxolitinib, a JAK inhibitor, blocks IFNβ-induced MCP-1 transcription. IFNβ induces STAT1 and STAT2 phosphorylation and STAT activation may explain the effects of IFNβ on MCP-1 expression (Fig. 5, [32]). STAT1 binding sites have previously been identified in the MCP-1 promoter [46,47]. Our data supports a role for STAT1 and/or STAT2 in the control of MCP-1 transcription, as an IFNβ mediated feedback loop drives STAT1 and STAT2 tyrosine phosphorylation in response to LPS. We have also shown that STAT1 is recruited to the *ccl2* promoter in response to IFNβ, this underlines the potential importance of STAT1 to MCP-1 transcription. STAT1 and STAT2 can form heterodimers [48,49] and therefore loss of STAT2 activation might be relevant to the loss of sustained MCP-1 production. In line with a potential role for STAT2, one study has shown that STAT2 KO mice produce less MCP-1 in a model of colitis [50]. A role for STAT6 in regulating MCP-1 expression in murine peritoneal macrophages has been suggested [51]. Our data however demonstrates that STAT6 phosphorylation is stronger in IFNαβR KO macrophages, which is consistent with a study showing that interferon β suppress STAT6 activation by IL-4 [52]. STAT6 is therefore unlikely to explain the regulation of MCP-1 by IFNβ downstream of TLR4 or 3 activation.

We show here that IL-10 can repress MCP-1 transcription, however the mechanism by which this occurs is not clear. One interpretation is that IL-10 directly represses MCP-1 transcription. Alternatively, this could be due to IL-10 repressing IFNβ production, as less IFNβ would limit the IFNβ-mediated feedback loop required to sustain MCP-1 transcription. IL-10 KO macrophages produce higher levels of IFNβ in response to LPS. Furthermore, addition of exogenous IL-10 results in decreased IFNβ secretion [35,36]. This increase in IFNβ could explain the elevated levels of MCP-1 transcription in LPS-stimulated IL-10 KO macrophages.

It has been previously published that the ERK1/2 pathway will negatively regulate IFNβ production [40], blocking the ERK1/2 pathway could therefore be predicted to lead to increased production of MCP-1 by enhancing the IFNβ autocrine loop. Whilst pharmacological inhibition of ERK1/2 did indeed cause enhanced IFNβ transcription, a resultant increase in MCP-1 production was not evident. In fact, loss of ERK1/2 signaling resulted in the reduction of MCP-1 mRNA induction and protein secretion at later time points, demonstrating the importance of ERK1/2 in MCP-1 transcription as previously published [17].

IFNβ is a key mediator of viral immune responses and so it is interesting that MCP-1 is directly up-regulated in response to IFNβ. Studies involving knockout mice for MCP-1 or its receptor, CCR2, show a role for this pathway in the anti-viral response. In a model of influenza pneumonia, WT mice have a profound increase in pulmonary MCP-1 levels. MCP-1 KO mice showed increased viral load, a diminished influx of macrophages and granulocytes and increased pro-inflammatory mediators such as TNFα and IL-6 [53]. MCP-1 and CCR2 deficient mice infected with murine CMV showed reduced accumulation of macrophages and NK cells as well as increased viral titers [54]. In West Nile virus infection, CCR2 deficiency results in reduced monocyte accumulation in the brain and increased mortality from encephalitis [55]. These studies show a direct role for MCP-1 in anti-viral responses and therefore the IFNβ mediated induction of MCP-1 downstream of TLR3 and/or 4 may important for an appropriate host response to viral infection.

In conclusion, we show that in macrophages the sustained production of MCP-1 in response to LPS or poly(I:C) requires an autocrine IFNβ signaling loop. In addition, we show that Ruxolitinib, a selective JAK inhibitor, can attenuate the transcription of MCP-1.
